# Development of a Serious Game App (Digimenz) for Patients With Dementia: Prospective Pilot Study for Usability Testing in Inpatient Treatment and Long-Term Care

**DOI:** 10.2196/69812

**Published:** 2025-10-27

**Authors:** Sören Freerik Brähmer, Benjamin Iffland, Stefan Kreisel, Martin Driessen, Eva M Trompetter, Meret Schomburg, Max Toepper, Carolin Steuwe

**Affiliations:** 1 Clinical Psychology and Psychotherapy Working Group, Department of Psychology, Bielefeld University, Universitätsstr 25Bielefeld, 33615, Germany, 49 (0)52110686604; 2 Department of Psychiatry and Psychotherapy, University Medical Center OWL, Evangelisches Klinikum Bethel, Bielefeld University, Bielefeld, Germany

**Keywords:** dementia, inpatient treatment, long-term care, nonpharmaceutical treatment, eHealth, serious games, pilot study, usability

## Abstract

**Background:**

In the face of an increasing treatment need among people with dementia, effective and efficient interventions with a focus on quality of life need to be established. In this context, serious games have received increasing attention. However, there is a lack of apps specifically designed for people with dementia.

**Objective:**

In this prospective pilot study, we examined the usability of a newly developed serious game app (“Digimenz”).

**Methods:**

A total of 43 people with cognitive impairment and mild to severe dementia completed the repeated-measures study procedure. Participants were recruited from an inpatient geriatric psychiatric ward and a long-term care facility. Participants were asked to complete 4 conditions in randomized order, including playing 3 different serious games (experimental conditions) and reading a newspaper (control condition). Each condition was completed once, and the total duration was 60 to 90 minutes per participant. Data on app usability were collected through self-ratings and observation after each condition. We tested for differences in usability among the conditions and the recruitment sites, and analyzed the relation of usability to cognitive capacity.

**Results:**

The serious games were accepted in both settings (long-term care: 30/30, 100% interested; psychiatric ward: 31/41, 76% interested), although study completion was lower in the psychiatric subsample (15/41, 37%) than in the long-term care subsample (28/30, 93%). Global usability was rated good (System Usability Scale global mean score: 79). More severely impaired patients had more pronounced difficulties in learning how to play the games (ρ_MMSE, Learnability_=−0.61, 95% CI −0.78 to −0.36; *P*<.001) and playing them alone (ρ_MMSE, Support_=−0.49, 95% CI −0.69 to −0.19; *P*<.001). Nevertheless, playing the games was associated with a more positive mood (likelihood ratio *χ*^2^_3_**=**25.09; *P*<.001), independent of the level of cognitive functioning (likelihood ratio *χ*^2^_1_**=**0.64; *P*=.42). All games were played with a moderate error rate (0.19‐0.49).

**Conclusions:**

Our results indicated a positive association between serious game usage and well-being in patients with dementia, given adequate support. This is a valuable addition to the understanding of serious game usage in dementia care. Although challenging, user-centered development of serious games with people who are severely impaired by dementia is an important research target. Limitations like low data quality and a simplified design are inherent in this study population. Nevertheless, we demonstrated how usability testing in this target group is possible through careful definition and operationalization. The inclusion of different data sources, different recruitment sites, and different levels of cognitive impairment increased the generalizability of the findings. To accommodate severely impaired patients, future developments should incorporate a broader range of difficulties and adaptations to group settings.

## Introduction

### Interventions in Dementia Care

The number of people living with dementia has been rising globally from 20.2 million in 1990 to more than 50 million in 2019 and is estimated to exceed 150 million in 2050 [1,2]. The rising prevalence leads to increasing numbers of patients with dementia who receive inpatient treatment or live in inpatient care facilities. It is important to provide suitable interventions for these patients. Possible intervention targets include improved quality of life, delayed cognitive decline, and successful treatment of comorbid disorders such as depression [3]. Given the broad range of dementia types and courses, these targets are not easy to address [4-7]. To satisfy patient needs, interventions are required to account for the various types of impairments and allow for individual tailoring. Accordingly, the Alzheimer Society of Canada [8] has put forward a person-centered approach as the best practice for dementia care, and dementia-friendly environments are receiving increasing attention [9,10]. In fact, nonpharmaceutical treatment (NPT) has become the recommended treatment for the behavioral and psychological symptoms of dementia [11]. NPT covers a wide range of approaches, including exercise and motor rehabilitation, cognitive rehabilitation, and psychological therapy, as well as information technology and assistive technology [12].

Unfortunately, the need for a person-centered and individually tailored approach puts a high burden on health care systems and private caregivers. Particularly in later disease stages, dementia causes larger health care challenges than other diseases [13]. This often conflicts with the already high costs, the lack of staff, and the short duration of inpatient treatment [14-16]. A possible approach to address these difficulties could be the use of serious games (SGs).

### SGs in the Treatment of Dementia

SGs have received increasing attention as a means to apply NPT in dementia treatment [17,18]. SGs are defined as games that are designed to not only entertain but also enhance learning, for example, in the area of psychoeducation or cognitive training [19]. There is a small but growing body of SGs being specifically targeted at people with dementia [20-26]. To accommodate the differential needs of this target group, different author groups propose development guidelines for SGs [27,28]. However, the number of studies involving SGs that meet these guidelines [20,29] or engage in systematic usability testing of SGs [30] is limited. Additionally, more severely impaired target groups are seldom thought of as potential SG users [18]. While unsupervised SG usage seems inherently less likely for people with severe dementia, high user satisfaction (ie, positive feelings toward SG usage) has been found to be independent of cognitive impairment under the premise of adequate app design [25,31]. Given their potential benefits as a delivery method for NPT, we argue that it is a valuable aim to develop SGs specifically for people with dementia, with consideration of severely impaired patients. Since the development of medical apps often tends to pay not enough attention to evidence-based standards [32], further studies are needed to establish utility estimates of SGs for patients with dementia to enhance clinical credibility [12].

### Usability of SGs for Patients With Dementia

This paper presents a pilot study of the usability of a newly developed SG (“Digimenz”) for older patients, promoting participative development. Usability in the context of dementia care has previously been operationalized by the ISO 9241 model [30,33]. It defines usability in terms of effectiveness, efficiency, and user experience. The ability to learn the handling of software is also a common part of different usability definitions [34]. While it is missing in ISO 9241, it appears to be a crucial factor for patients with dementia who have limited learning capacities by definition. Therefore, we have followed Abran et al [35] who propose to augment ISO 9241 by the concept of learnability and use this as an integrative model of usability (Table 1).

**Table 1.** Definition of usability.

Construct	Definition
Effectiveness	Achieve the given aims with precision and completeness
Efficiency	Resources needed in relation to the results achieved
User experience	Perceptions, emotions, and attitudes related to software use, for example, user satisfaction or interest
Learnability	Time needed to learn the handling of the software

### Rationale of the Study

The purpose of this study was to generate a feedback loop for the iterative and evidence-based development of an SG app by providing scientific testing and making the results available for further development of this and other SG apps. This will add to a diverse, person-centered, and resource-saving body of intervention options for patients with dementia.

To ensure that the full spectrum of cognitive impairment is covered when examining the usability of the SG app, we recruited participants from two settings: (1) a long-term care residential facility (where people live with, on average, a lesser degree of cognitive impairment) and (2) a geriatric psychiatric inpatient ward (where the degree of deficits is expected to be more severe). We examined whether the Digimenz app is usable across different degrees of impairment while controlling for the effects of the recruitment site.

We were particularly interested in whether the SG shows sufficient usability in terms of global usability, effectiveness, efficiency, user experience, and learnability. We hypothesized that less impaired patients would report higher overall usability of the SG app and show higher effectiveness, efficiency, and learnability, but would have an equally satisfying user experience as patients with a higher degree of impairment. Moreover, usability parameters are expected to be similar in the psychiatric context and in a nursing home.

## Methods

### Participants

Following the adapted CONSORT (Consolidated Standards of Reporting Trials) statement for pilot trials [36], the sample size was adapted to the rationale of usability testing. To our knowledge, there is no evidence regarding the effect size for the comparison of the usability parameters of SGs among users with different levels of cognitive impairment. We therefore chose a sample size comparable to that in other usability trials involving people with cognitive impairment [30] in order to allow reliable conclusions about SG usability and inform future large-scale intervention studies. The final sample included 43 participants.

Subsample 1 (n=28) was recruited from a nursing home for older adults specialized in the management of cognitive impairment. Residents are referred to this kind of long-term care facility when care in their original homes is no longer adequate or possible. Subsample 2 (n=15) was recruited from 2 geriatric-psychiatric wards specialized in dementia management in a psychiatric hospital. Patients are referred to this kind of psychiatric ward in case of acute exacerbation of mental impairments or comorbid mental disorders.

The inclusion criterion for all participants was documented cognitive deficits (mild cognitive impairment [MCI] or dementia) diagnosed at admission to the respective institution by a specialist in the field according to the criteria by McKhann et al [37] and Albert et al [38]. Further, participants were included only if they had sufficient German language proficiency. Since we wanted to represent a broad range of cognitive impairments, we tuned the inclusion criteria for sensitivity and not specificity. Therefore, a specific etiology of cognitive impairment was not relevant for inclusion. Moreover, we did not define age limits, numerical cutoffs for cognitive tests, or comorbid mental disorders as exclusion criteria.

### Ethical Considerations

The study protocol complies with the Declaration of Helsinki and received ethical approval from the Bielefeld University Ethics Review Board (EUB-2022‐265 s). Potential participants were approached to examine their explicit preferences regarding study participation. The purpose and methodology of the study were explained in simple terms to be accessible for those who were nominally more severely cognitively impaired. If there was any sign of rejection, no further recruitment was attempted. If a potential participant expressed interest in participation, informed consent was acquired. To this end, an information sheet and consent form were read and signed by the participant, if mental capacity allowed it. Otherwise, informed consent was obtained from a legal representative or relative. Study data were deidentified and saved on the secure servers of our institution. To guarantee the anonymity of participating patients, participant-related data will not be made available. No compensation was provided.

### Study Design

In this prospective study, we used a repeated-measures design. Each participant was asked to complete 4 conditions in randomized order. Three of these conditions involved playing 3 different games from the SG app (“Digimenz”). The fourth condition included reading a newspaper serving as a naturalistic control condition. All conditions consisted of 2 runs each (5 min), with one supported by the conducting researcher and the other performed with minimal support. Prior to the experiment and after each individual condition, the participants answered a set of questions and the conducting researcher gave external ratings. Additional unstructured qualitative feedback was registered. Global cognitive functioning was assessed in a separate session. The total duration for each participant was about 60‐90 minutes. To minimize researcher bias while retaining a high data quality, data collection was performed by 2 research assistants who were instructed and supervised by the principal investigators (MT, EMT, and SK), who have extensive experience in dementia research. The study has been registered in the German Clinical Trials Register (DRKS00031363).

### Intervention

#### Digimenz App Overview

The Digimenz app is a commercial tablet app linked to research through a European Union–funded project (IT4Anxiety). The project’s objective is to connect end users of e-mental health apps, scientists, and industry professionals, providing a platform for codevelopment, testing, and implementation of innovative treatment options. The Digimenz app has been developed in close cooperation with people affected by dementia and their caregivers. They were involved in an iterative, user-centered process of concept development, prototype testing, feedback collection, and software development until deriving the SG app tested in this study. Three games have been incorporated in the Digimenz app, all of which require psychomotor integration: recognizing objects and moving them by drag and drop (“Drag&Drop”), recognizing distorted pictures (“Object Recognition”), and sorting letters into words (“Spelling”). The difficulty of the games is adjusted automatically within 4 levels per game (higher levels entail higher difficulty). The level can change dynamically during a gaming session. It is determined through a running score, which is calculated based on the user’s current response time and error rate. The score is updated once per second and after each user input. Dynamic difficulty adjustment is a central recommendation for making SGs usable for patients in different stages of cognitive decline [27]. Further guidelines from Eichhorn et al [28] were considered regarding the design and timing of assignments, help, and feedback. The motor requirements for the games include dragging and tapping. Therefore, hand-eye coordination is required, but no complex, multistep, or whole-body movements are involved. The different SGs relate to different cognitive subfunctions: all games require semantic memory, working memory, and executive functioning. The Drag&Drop game additionally requires motor functioning and visuospatial functioning. The Spelling game requires lexical functioning. Therefore, we did not compute mean values of usability measures across the games but analyzed usability for the individual games separately.

#### Drag&Drop Game

**Figure 1.**
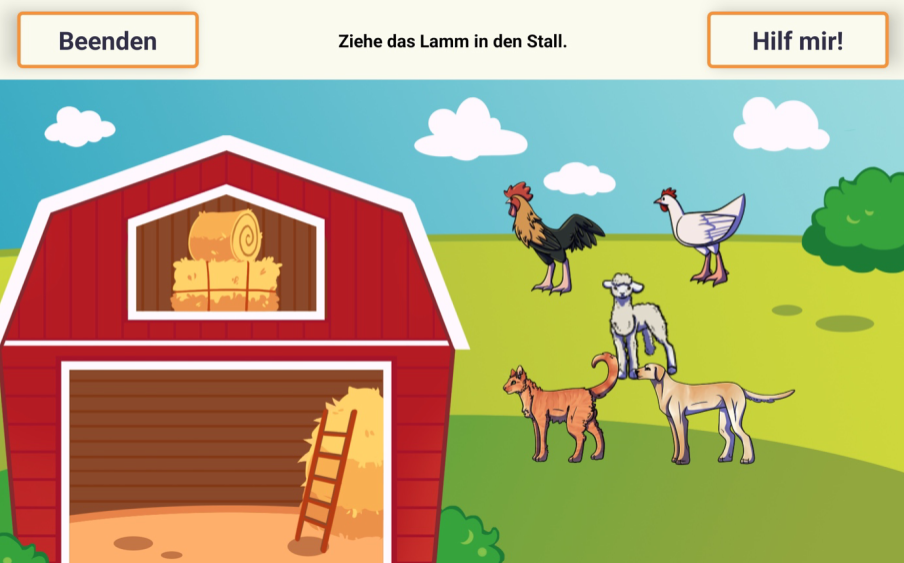
Example screen of the Drag&Drop game in the Digimenz app.

In this SG, animals must be sorted in a stable. The screen presents a choice of animals (targets and distractors) on a meadow, next to an empty stable (Figure 1). An assignment is given (eg, “Put the lamb in the stable”), and the users must choose the correct animal, place their finger on it, and place it in the stable via drag and drop. A higher difficulty is achieved by presenting more distractors and by giving more abstract assignments (eg, “Put all animals with hooves in the stable”). If an incorrect animal is placed in the stable, it disappears from the screen, and a red cross is presented (counted as an error). If a correct animal is placed in the stable, a green checkmark is presented, and the animal remains in the stable. The task cannot be skipped but is solved by moving all animals into the stable.

#### Object Recognition Game

In this game, a picture of an animal is presented, but it is covered by multiple tiles. The tiles disappear slowly, making it easier to recognize the picture until the full picture can be seen. Next to the picture, multiple animal names are presented as buttons (one target and one or multiple distractors). The assignment is to tap on the animal name that matches the picture. Difficulty is varied by changing the number of distractors and by altering the speed at which the tiles disappear. If a wrong name is chosen, it turns red to distinguish it from the remaining choices (counted as an error). The task cannot be skipped, but the distractors disappear after some time, such that only the right answer remains.

#### Spelling Game

In the third game, a picture of an animal is presented. In the lower part of the screen, the letters needed to spell the name of the animal are presented in randomized order. The task is to tap on the letters in the correct order to spell the name of the animal. If a wrong letter is chosen, it blinks in red (counted as an error). Difficulty is influenced by providing longer animal names. The task cannot be skipped but must be solved by tapping the letters presented on the screen in random order until discovering the right order by chance.

#### Control Condition

To control whether changes in mood would be attributable to SG usage as opposed to interaction with the researcher or study participation, we added an illustrated newspaper as a naturalistic control condition. It can be used in one-to-one interaction with varying amounts of support and is thus comparable to the SGs from the Digimenz app. Depending on the usage style (looking at pictures, reading headlines or short texts, and reading longer texts), it can accommodate different degrees of cognitive impairment.

### Measures

To test the applicability of the SGs, we recorded reasons for nonparticipation in the recruitment phase and dropout thereafter. To test usability and its relation to cognitive functioning, as well as differences between the subsamples, we operationalized global usability and the individual usability constructs as follows:

- Global usability: The System Usability Scale (SUS) [39] (total score 0‐100) was assessed after completing all 4 conditions. We used a simplified version for people with cognitive impairment [40]. SUS scores can be classified by comparing them to the existing literature. Since no specific norms for SGs exist, we used the universal norms defined by Sauro and Lewis [41], who stated that a SUS score of 68 denotes average usability (school grade C) and good usability can be assumed for SUS scores higher than 74 (school grade B).
- Effectiveness: We used the error rate in the second run of each SG to operationalize their correct usage. Multiple errors could be made per task, and therefore, the error rate can be greater than 1. We also collected data on the highest level reached by each participant to allow better interpretation of error rates.
- Efficiency: We used the amount of support needed in the minimal-support run (second run of each condition; 0 [“no support needed”] to 5 [“much support needed”]) as indicated by the researcher to operationalize the efficiency of the intervention. Scores were based on a scoring guideline that required information about the type, intensity, and frequency of support.
- User experience: At baseline and after each condition, the investigator and the participant rated the participant’s mood (positive or negative affect) using a visual analog mood scale (VAMS) [42]. The researcher provided their own rating before asking the participant for their rating.
- Learnability: The item “Playing the game alone is too difficult for me” was answered by the participant on a 5-point Likert scale after each condition. The researcher answered the item “The game is too difficult for the participant” on the same scale after each condition. In addition, the proportion of participants needing support (yes or no) was compared between the first and second runs of each condition.

Efficiency, user experience, and learnability were compared between the SGs and the control condition (newspaper). Sufficient usability in the different settings was assumed in the case of noninferiority of these parameters when comparing the SGs to the control condition. A “good” global usability rating (SUS scores >74) was the second indicator of sufficient usability. Error rate cannot be used in this way since it is not defined for the control condition. In addition to these quantitative measures of usability, we registered any additional feedback on usability issues given by the participants during SG usage and while rating the scales on usability. Global cognitive functioning was assessed with the Mini-Mental State Examination (MMSE) [43]. Demographic information (age and sex) and any existing diagnoses were registered from the medical record.

### Quantitative Data Analysis

We present descriptive statistics of the rates of nonuse and dropout as operationalization of the applicability of the SGs. We modeled the usability measures according to the conditions and the degree of cognitive impairment (MMSE) and their interaction, which we hypothesized to be the most relevant predictors of usability. We controlled for the effects of the recruitment site (subsample), given the differences between the 2 sites. We used mixed effect models (linear mixed models for continuous outcome variables and cumulative link mixed models [CLMMs] for ordinal outcome variables) with random intercepts to account for the repeated-measures design. These models have the advantage of using all available data at each measurement, precluding the need for listwise deletion (ie, a person does not have to be deleted if missing 1 measurement). Nagelkerke pseudo *R*^2^ is reported as an effect size measure by comparing the complete model to the model without the respective predictor and its interaction terms. To quantify differences between conditions, we computed Hedges *g* using the within-subject variance [44] and the adjustment for small samples: $g=\frac{M_{condition\;a}-M_{condition\;b}}{SD_{diff}}*\sqrt{2(1-r_{condition\;a,\;condition\;b}}*(1-\frac{3}{4n-5})$ [45]. Values of g >0.2 may be interpreted as small, *g*>0.5 as medium, and *g*>0.8 as large effect sizes [44]. All analyses were performed in R version 4.4.1 (R Foundation for Statistical Computing). Linear mixed models were fitted with the lme4 package [46], and CLMMs were fitted with the ordinal package [47]. The significance level was set to *α*=.05 for all inferential tests.

### Qualitative Analysis of Feedback

Qualitative feedback data on usability issues were subjected to a thematic content analysis following Kuckartz and Rädiker [48]. This is a multistep, iterative procedure. The steps are as follows:

1. Initial work with the data: Feedback and field notes were reviewed, annotated, and summarized (authors SFB and MS).
2. Deductive development of main categories: We predefined categories for the 3 SGs to capture any feedback specific to one of the SGs (authors SFB and MT).
3. Coding of data with main categories: The complete qualitative dataset was coded as far as possible with the predefined categories (author SFB).
4. Inductive development of further categories: Based on the first coding process, we defined further categories to account for themes not covered by the predefined categories (authors SFB and MT).
5. Recoding of data with all categories: The complete qualitative dataset was coded again, using both deductive and inductive categories (author SFB).
6. Interpretation and presentation: The results were summarized into a concise report (authors SFB, MT, and CS).

Steps 4 and 5 were repeated several times to fine-tune the categories.

## Results

### Sample Characteristics and Acceptability of the Intervention

**Figure 2.**
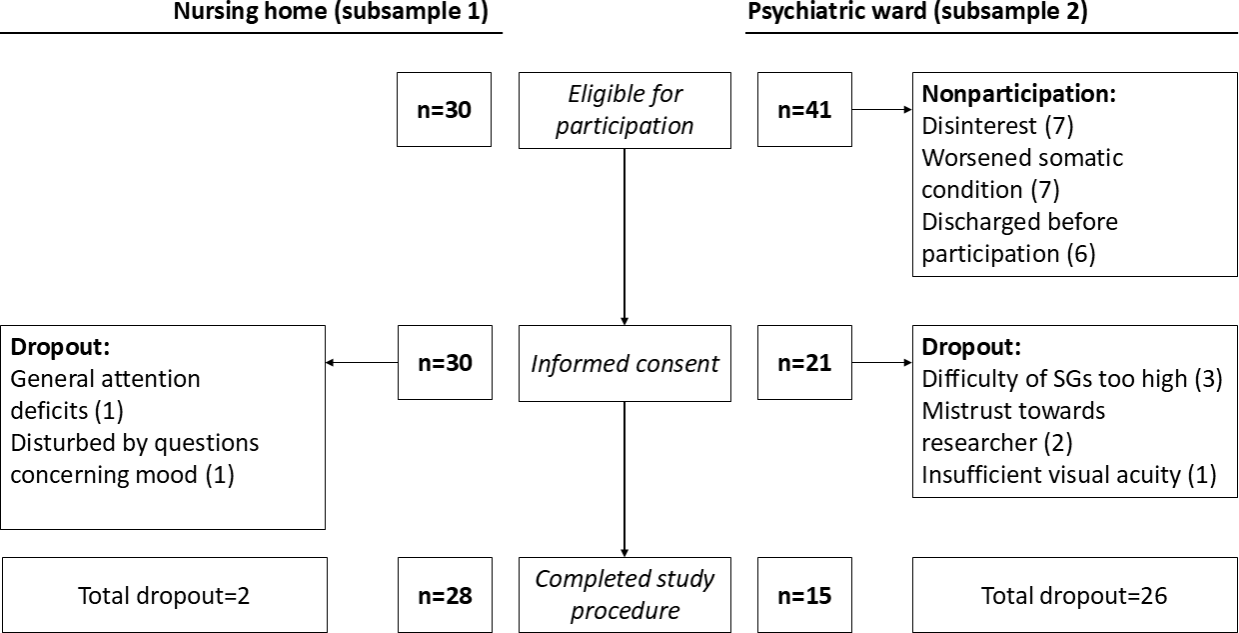
Recruitment flowchart. SG: serious game.

In subsample 1 (nursing home), of 30 eligible individuals, 30 (100%) consented to participate and 28 (93%) completed the study procedure. In subsample 2 (psychiatric ward), of 41 patients eligible for participation, 21 (51%) consented to participate and 15 (37%) completed the study procedure. Table 2 details the characteristics of both samples. Figure 2 provides an overview of the reasons for nonparticipation and dropout. In subsample 1, all people approached were interested in the SGs, and the reasons for dropout were not specific to the games themselves. In subsample 2, 10 people (24%) denied participation, which was specifically related to SG usage (7 [17%] were disinterested, and 3 [7%] were frustrated by the SGs), while 31 people (76%) were generally interested in the SGs. The odds ratio for dropout between informed consent and study completion was 5.6 (*P*=.05), that is, the odds of dropping out of the study were 5.6 times higher in the psychiatric ward subsample than in the nursing home subsample.

**Table 2.** Sample characteristics.

Characteristic	Nursing home (subsample 1) (N=28)	Psychiatric ward (subsample 2) (N=15)
Age (years)		
Mean (SD)	85.6 (7.3)	80.2 (7.5)
Range	57-97	68-94
Cognitive functioning (MMSE^a^ score)		
Mean (SD)	20.1 (6.4)	14.7 (6.1)
Range	4-28	4-22
Sex (female), n (%)	22 (79)	9 (60)
Formally diagnosed with dementia (yes), n (%)	11 (39)	15 (100)
Main diagnoses, n (%)		
Dementia due to Alzheimer disease with late onset	1 (4)	2 (13)
Unspecified dementia, unspecified severity	10 (36)	6 (40)
Multicause dementia due to Alzheimer disease and vascular disease	—^b^	4 (27)
Vascular dementia, subcortical	—	2 (13)
Delirium superimposed on dementia	—	1 (7)

a MMSE: Mini-Mental State Examination; measured on a scale from 0‐30.

b Not applicable.

### Global Usability and Usability Dimensions

To compute the total SUS score, missing values were replaced by the scale mean, as recommended by Lewis [49]. In the psychiatric subsample, the SUS total score was 71.5 (SD 24.33), while in the nursing home subsample, the SUS total score was 85.10 (SD 12.67). Less cognitive impairment was associated with better usability ratings in both subsamples, with no significant difference in the correlation coefficients between the subsamples (total sample: Spearman ρ=0.31; *P*=.03). The results for the individual usability dimensions as defined in Table 1 are presented for the total sample in Table 3. Differences between the subsamples were tested in the following multilevel analyses.

**Table 3.** Descriptive statistics for usability dimensions (total sample).

Usability dimension and operationalization		Condition								MMSE^a^^,^^b^		
		Newspaper		Drag&Drop		Object Recognition		Spelling		*r*^c^ (95% CI)	ρ^d^ (95% CI)	*P* value
		Mean (SD)	*g* ^e^	Mean (SD)	*g*	Mean (SD)	*g*	Mean (SD)	*g*			
Effectiveness												
Error rate		—^f^	—	0.35 (0.40)	—	0.19 (0.25)	−0.43	0.49 (0.43)	0.38	−0.14 (−1.00 to 0.13)	—	.20
Efficiency												
Amount of support in run 2 (0-5)		1.36 (2.10)	—	1.43 (1.82)	0.04	1.16 (1.62)	−0.11	1.79 (1.90)	0.21	—	−0.49 (−0.69 to −0.19)	<.001
User experience												
Mood (self-rated; 1-5)		3.36 (0.90)	—	4.00 (0.75)	0.78	3.85 (0.85)	0.55	3.78 (0.86)	0.46	—	0.07 (−0.31 to 0.41)	.67
Mood (externally rated; 1-5)		3.44 (0.63)	—	3.85 (0.69)	0.60	3.85 (0.85)	0.61	3.74 (0.73)	0.46	—	0.12 (−0.18 to 0.41)	.45
Learnability												
Too difficult (self-rated; 1-5)		1.98 (1.35)	—	1.51 (0.98)	−0.38	1.48 (0.99)	−0.41	1.71 (1.04)	−0.21	—	−0.15 (−0.44 to 0.17)	.18
Too difficult (externally rated; 1-5)		2.42 (1.53)	—	2.24 (1.56)	−0.12	1.98 (1.32)	−0.31	2.37 (1.41)	−0.04	—	−0.61 (−0.78 to −0.36)	<.001
Need for support (% yes in run 1)		33 (48)	—	65 (48)	0.65	53 (50)	0.40	74 (44)	0.87	−0.61 (−1.00 to −0.42)	—	<.001
Need for support (% yes in run 2)		32 (47)	—	44 (50)	0.23	40 (49)	0.14	58 (50)	0.51	−0.48 (−1.00 to −0.25)	—	<.001

a MMSE: Mini-Mental State Examination.

b Correlation coefficients denote the correlation of the respective variable with the MMSE score across the 3 serious game conditions.

c Pearson *r*.

d Spearman ρ.

e For error rate, each condition is compared to the Drag&Drop condition; for all other variables, each condition is compared to the newspaper condition. Moreover, *g*>0.2 indicates a small effect, *g*>0.5 indicates a medium effect, and *g*>0.8 indicates a large effect.

f Not applicable.

### Effectiveness

No significant effects of MMSE (*F*_1,36.50_=0.10; *P*=.75; *DR*^2^=0.09) and subsample (*F*_1,36.45_=0.16; *P*=.69; *DR*^2^<0.001) on error rate were found in the multilevel analysis. Among the SGs, there were significant differences in error rates (*F*_2,74.28_=9.43; *P*<.001; *DR*^2^=0.31; Object Recognition game: −0.10; *P*=.09; Spelling game: +0.15; *P*=.01; see Table 3 for effect sizes). The interaction of MMSE and condition was not significant (*F*_2,74.02_=0.13; *P*=.15; *DR*^2^=0.09). The Nagelkerke *R*^2^ for the complete model compared to the null model was *R*^2^=0.37. Regarding the difficulty adjustment, Table 4 shows a descriptive analysis of the difficulty levels. Overall, the highest (ie, most difficult) level was seldom reached, and participants with a higher MMSE reached higher levels of difficulty.

**Table 4.** Participants per difficulty level and association with the MMSE^a^.

Condition (game)	Level				ρ^b^	*P* value
	1^c^, n (%)	2^c^, n (%)	3^c^, n (%)	4^c^, n (%)		
Drag&Drop	21 (49)	9 (21)	10 (23)	3 (7)	0.58	<.001
Object Recognition	16 (37)	15 (35)	8 (19)	4 (9)	0.47	.002
Spelling	28 (65)	14 (33)	1 (2)	—^d^	0.46	.003

a MMSE: Mini-Mental State Examination.

b Spearman ρ showing the association between the MMSE and the maximum level reached.

c Number and proportion of participants who reached no higher than this level.

d Not applicable.

### Efficiency

**Figure 3.**
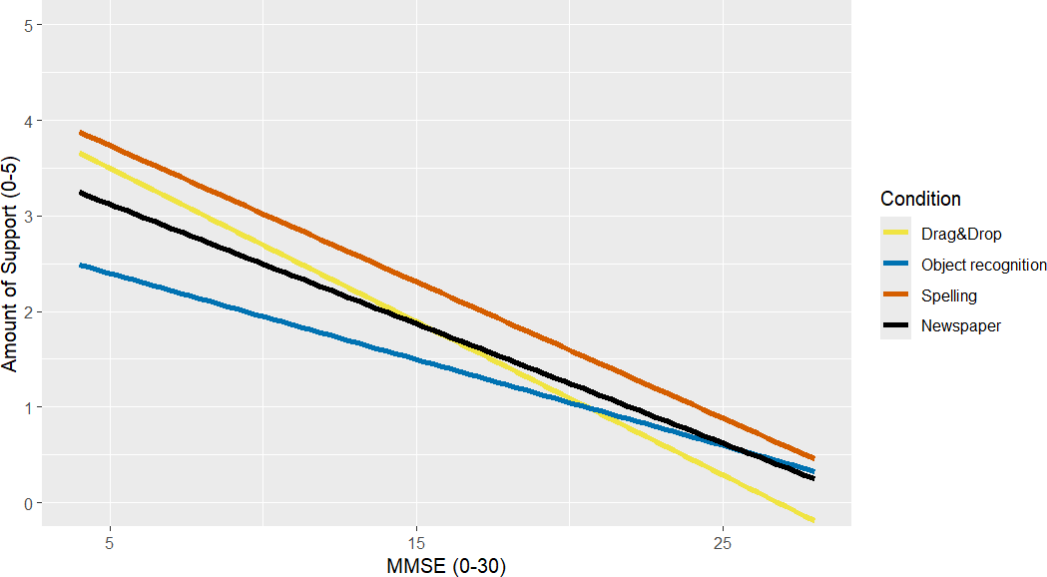
Association between support needed and Mini-Mental State Examination (MMSE) scores in all experimental conditions.

Participants with a higher MMSE needed significantly less support than those with a lower MMSE (Table 5; Figure 3). The correlation analysis presented in Table 3 suggests that this effect was of medium size (ρ_MMSE, Amount Support_=−0.49). For the Spelling game, significantly more support was needed than for the newspaper condition (+1.27; *P*=.02). This was not the case for the Drag&Drop game (+0.17; *P*=.77) and the Object Recognition game (−0.09; *P*=.88) (see Table 3 for effect sizes). No significant differences between the subsamples were found in the multilevel analysis. The Nagelkerke *R*^2^ for the complete model compared to the null model was *R*^2^=0.16.

**Table 5.** ANOVA for the amount of support (cumulative link mixed model)^a^.

Predictor	LR^b^ chi-square (*df*)	*P* value	Δ*R*^2^
Condition	10.32 (3)	.02	0.07
Subsample	0.90 (1)	.35	0.006
MMSE^c^	13.60 (1)	<.001	0.09
Condition*MMSE	1.56 (3)	.67	0.01

a Model formula: amount of support ~ 1 + condition + sample + MMSE + condition*MMSE + (1|subject).

b LR: likelihood ratio.

c MMSE: Mini-Mental State Examination.

### User Experience

**Figure 4.**
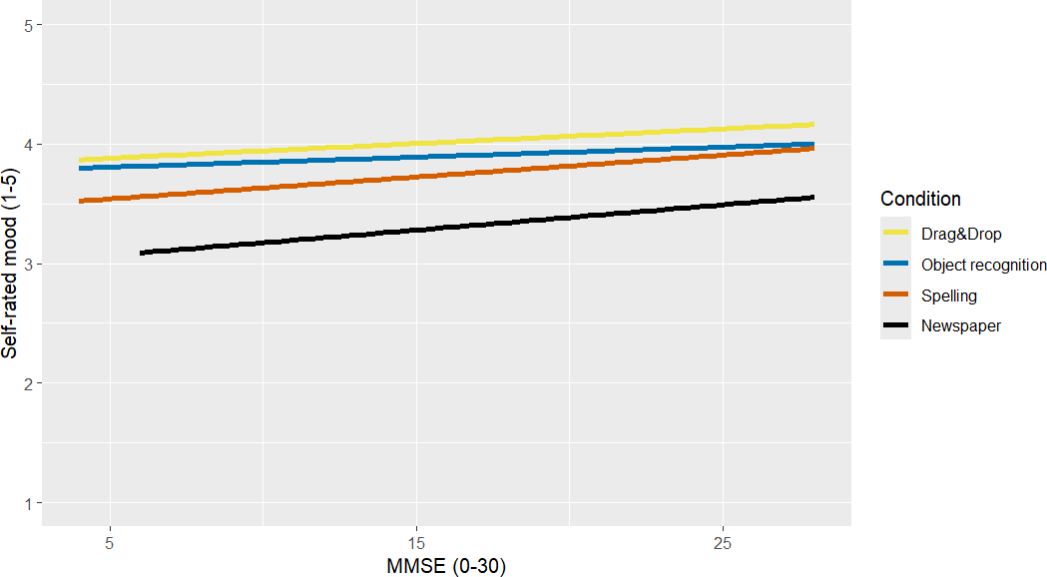
Association between self-rated mood and Mini-Mental State Examination (MMSE) scores in all experimental conditions.

The multilevel analysis showed that self-reported mood was higher in all 3 game conditions than in the newspaper condition (Drag&Drop: +2.55; *P*<.001; Object Recognition: +2.17; *P*<.001; Spelling: +1.56; *P*=.004; see Table 3 for effect sizes), when correcting for baseline mood (Table 6). The MMSE was not significantly correlated with self-reported mood (Table 6; Figure 4). There were no significant differences between the 2 subsamples. The Nagelkerke *R*^2^ for the complete model compared to the null model was *R*^2^=0.24. The external rating of mood showed a similar pattern with higher mood ratings in all 3 game conditions compared to the newspaper condition (Drag&Drop: +1.65; *P*<.001; Object Recognition: +1.28; *P*=.007; Spelling: +1.08; *P*=.02; see Table 3 for effect sizes). The overall correlation of self-reported and externally rated mood was *r*=0.64 (95% CI 0.43-0.79; *P*<.001).

**Table 6.** ANOVA for self-reported mood (cumulative link mixed model)^a^.

Predictor	LR^b^ chi-square (*df*)	*P* value	Δ*R*^2^
Condition	25.09 (3)	<.001	0.17
Baseline mood	9.76 (1)	.002	0.07
Subsample	0.70 (1)	.40	0.005
MMSE^c^	0.64 (1)	.42	0.005
Condition*MMSE	0.81 (3)	.85	0.006

a Model formula: self-rated mood ~ 1 + condition + sample + MMSE + condition*MMSE + baseline mood + (1|subject).

b LR: likelihood ratio.

c MMSE: Mini-Mental State Examination.

### Learnability

According to the multilevel analysis (Table 7), self-rated difficulty was independent of the MMSE (estimate=−0.06; *P*=.54). Compared to the newspaper condition, it was significantly lower for the Drag&Drop game (−2.01; *P*=.002) and Object Recognition game (−1.76; *P*=.005), but not for the Spelling game (−0.63; *P*=.54; see Table 3 for effect sizes). The 2 subsamples did not differ significantly in their difficulty ratings. The Nagelkerke *R*^2^ for the complete model compared to the null model was *R*^2^=0.14. The external rating of difficulty, in contrast, showed a strong negative association with MMSE (estimate=−0.22; *P*<.001) (ie, higher MMSE scores were associated with lower external ratings of difficulty). The overall correlation of self-reported and externally rated difficulty was ρ=0.52 (95% CI 0.25-0.72; *P*<.001).

**Table 7.** ANOVA for self-rated difficulty (cumulative link mixed model)^a^.

Predictor	LR^b^ chi-square (*df*)	*P* value	Δ*R*^2^
Condition	12.05 (3)	.007	0.08
Subsample	2.27 (1)	.13	0.02
MMSE^c^	0.67 (1)	.41	0.005
Condition*MMSE	3.39 (3)	.29	0.03

a Model formula: self-rated difficulty ~ 1 + condition + sample + MMSE + condition*MMSE + (1|subject).

b LR: likelihood ratio.

c MMSE: Mini-Mental State Examination.

Our second indicator of learnability was the proportion of participants needing support in the full-support run versus the minimal-support run (Multimedia Appendix 1).

In the full-support run, more participants needed support in the 3 game conditions than the newspaper condition (Drag&Drop: +0.28; *P*<.001; Object Recognition: +0.19; *P*=.02; Spelling: +0.41; *P*<.001; see Table 3 for effect sizes; *F*_3,275.18_=10.93; *P*<.001; *DR*^2^=0.14). From the full-support run to the minimal-support run, the rate of support decreased significantly (*F*_1,274.92_=9.86; *P*=.002; *DR*^2^=0.05). Accordingly, there were no significant differences in support needed between the game conditions and the newspaper condition in the second run (Drag&Drop: −0.20; *P*=.09; Object Recognition: −0.11; *P*=.34; Spelling: −0.16; *P*=.17; see Table 3 for effect sizes). The interaction effect of run and condition was not significant (*F*_3,274.9_=1.09; *P*=.36; *DR*^2^=0.02). Participants with a higher MMSE score were less likely to need support at all (*F*_1,37.89_=16.76; *P*<.001; *DR*^2^=0.07). There were no significant differences between the 2 subsamples (*F*_1,37.88_=0.004; *P*=.95; *DR*^2^ <0.001). The Nagelkerke R^2^ for the complete model compared to the null model was R^2^=0.25.

### Qualitative Feedback Data

The results of the thematic content analysis [48] of usability issues mentioned during SG usage and data collection are presented in Table 8. We chose to categorize usability issues according to the source they were attributed to. Besides usability issues specific to the SGs, participants also mentioned problems with the handling of the tablet and personal impairment that hindered tablet or SG usage.

**Table 8.** Thematic analysis of usability issues (verbal feedback).

Main category and subcategory		Usability issues
Usability issues attributed to personal impairment		
Sensory and motor problems		Impaired vision (n=2; MMSE^a^ scores of 16 and 26) Motor problems due to Parkinson disease (n=1; MMSE score of 17)
Cognitive problems		Concentration deficits (n=4; MMSE scores of 14, 15, 17, and 27) Tired (n=1; MMSE score of 18)
Usability issues attributed to tablet usage		Difficulties handling the tablet (eg, difficult to use the touchscreen) (n=3; MMSE scores of 4, 14, and 22) Eyes hurt after looking at the tablet for a while (n=1; MMSE score of 26)
Usability issues attributed to serious games		
All serious games		Reinforcement from the app not strong enough (n=1; MMSE score of 22) Computer-animated voice hard to understand (n=1; MMSE score of 16) Serious games too easy/repetitive (n=4; MMSE scores of 18, 24, 27, and 27)
Drag&Drop		Too many stimuli, cannot focus attention (n=1; MMSE score of 19) Difficulties distinguishing animals with similar shape (eg, cat and dog) (n=4; MMSE scores of 4, 14, 17, and 19) Motion exhaustive for the arm (n=1; MMSE score of 15) Instructions not understood (n=1; MMSE score of 18)
Object Recognition		Animals not recognized (n=2; MMSE scores of 9 and 9)
Spelling		Animals not recognized (n=4; MMSE scores of 4, 20, 22, and 26) Individual animals not known (n=1; MMSE score of 21) Difficulties with spelling (n=2; MMSE scores of 22 and 25)

a MMSE: Mini-Mental State Examination.

## Discussion

### Principal Findings

This study aimed to evaluate the usability of SGs for older adults with cognitive impairment, ranging from MCI to mild and severe dementia. We hypothesized that the SGs would be applicable for a nursing home as well as a psychiatric ward, that the usability would be sufficient, and that it would be differentially influenced by the level of cognitive impairment.

Most people approached were open to testing the SGs; however, a considerably smaller proportion managed to complete the study procedure in the psychiatric ward subsample, given greater impairment there. The global usability was rated good (SUS), and the indicators for individual usability dimensions showed that using the SGs was possible with a moderate error rate and support. Moreover, the support rate decreased from the first to the second usage, implying a learning process, and the gaming experience was associated with positive mood. While the amount of support needed and the ability to learn the handling of the games were related to cognitive impairment, the error rate and mood state after playing were not.

### Comparison With Prior Work

#### Applicability

The SGs can be considered applicable in both recruitment settings, with the restriction that study participation and openness to the SGs would be lower in the hospital context, owing most likely to the higher proportion of severely impaired participants [50]. One aspect is cognitive impairment, and importantly, nonparticipation, study dropout, and usability issues within the psychiatric subsample were attributed by several participants to somatic conditions and sensory and motor impairments. Moreover, the time it took to acquire informed consent from legal advisors sometimes precluded participation before discharge or transfer to another ward. While this is partly due to research requirements, quick patient turnover may generally limit the applicability of NPTs in routine care. Only 7 patients denied participation due to disinterest. To understand their specific reasons for nonparticipation, their internal experiences would need to be investigated. This, however, is difficult as more severe impairments are inversely related to the capacity of objectifiable communication. Moreover, the validity of questionnaire data collected from the patients is uncertain due to limited understanding of the items, and proxy ratings may be subject to bias [51]. It is therefore not surprising that most samples in SG usability studies are composed mainly of persons with MCI or mild dementia (MD) [30]. These user groups are mostly found to be using mobile devices and SGs successfully [18,52,53], which is in line with the high participation rate in our nursing home subsample (less affected). Encouragingly though, we did not find any significant differences in usability between the recruitment sites. This result is in line with our hypothesis of sufficient and similar usability in both subsamples.

Nevertheless, outside of standardized study procedures, people with MCI or dementia report extensive difficulties and reservations concerning mobile devices [54,55]. Therefore, to raise the acceptability of SGs and aid their uptake into routine care, motivational strategies may be helpful not only within the SGs but also to initiate usage [28]. Possible strategies may include social support or social referencing [56]. For example, future applications of the Digimenz SGs might benefit from adaptation to a group setting. This would facilitate the resource-efficient allocation of professional support and mutual support among participants [57,58]. Social interaction itself is also associated with increased well-being and reduced cognitive decline [59,60]. At the same time, caregivers would need to be trained to determine an appropriate level of support in individual and group settings. Another challenge may be the availability of devices. As in this case, SGs should be developed with an emphasis on compatibility across devices and operating systems. This reduces the need for specialized equipment and lowers the entry threshold for providers and users.

#### Usability

Regarding the SGs themselves, the global usability rating showed a positive correlation with the level of cognitive functioning (MMSE). This was reflected in difficulties in understanding the SGs and playing them without support (learnability and efficiency), which are related to cognitive impairment. These findings are in line with our hypotheses. Beyond global cognitive functioning, qualitative feedback data suggested several usability issues specific to the SGs. In the Drag&Drop game, several participants mentioned having difficulties distinguishing the animals, potentially hindering effective usage. In the Spelling game, the combined requirement of recognizing animals and spelling their names seemed to be especially demanding. Accordingly, quantitative estimates of effectiveness, efficiency, and learnability were least favorable for the Spelling game. The Object Recognition game, in contrast, was least demanding, according to quantitative and qualitative findings. For all 3 SGs, the support rate decreased from the first to the second run, which may indicate a learning process. Häikiö et al [61] showed that older users could significantly improve their interaction with a touch-based device after just 1 contact. Evidently, there remained a large proportion of participants who needed support in the second run, although not significantly more than in the newspaper condition. The qualitative analysis further suggested that several usability issues arose from factors not specific to the SGs, such as sensory and motor impairment, as well as general problems with handling the tablet. Although the SGs used here have already taken into account many of the usability recommendations given by Bouchard et al [27], they will need further adjustments to accommodate such a diverse user group as ours. Easier as well as more difficult levels would be needed. Improvements to in-game help and the user interface may include clearer contrast, better distinguishable shapes, visual clues, adjustable text and button sizes, broader use of multisensory feedback, and a refined voice guide.

The difficulties in using the SGs do not seem to be associated with the enjoyment taken from playing (user experience), since mood was consistently higher in the 3 game conditions than in the newspaper condition, irrespective of the cognitive status. This is again in line with our hypotheses. However, the study design does not allow for causal interpretation. Moreover, the differences in support given during the full-support run between the SGs and the newspaper condition may be a confounding factor. High levels of fun or enjoyment were also found in other studies evaluating SGs [20,31,62,63]. In some studies, healthy controls found the games more enjoyable than people with dementia or MCI [25,64]. Two studies assessing SGs specifically designed for people with dementia reported less enjoyment among healthy or less impaired participants, since they found the games too easy [20,63]. Here, 4 participants explicitly stated that they found the SGs too easy, most of whom had relatively little cognitive impairment. Contrasting to our hypothesis, we found no significant relation between the error rate and the level of cognitive functioning. This is probably due to the adaptive difficulty of the games and the support at hand. Less impaired participants reached more difficult levels of the games, while more severely impaired patients received more support. This may have equalized the error rates and the self-ratings of difficulty across levels of cognitive impairment.

### Strengths

It is a strength of this study that we chose theory-based operationalization of usability, enabling us to draw differentiated conclusions regarding the usability of the SGs and the relation to cognitive functioning. By using different data sources (external and internal ratings, qualitative data, and usage data from the SGs), we can provide comprehensive insights into the usage behavior and user experience. The diverse nature of our sample in terms of cognitive impairment and care settings allows us to set our results in the context of previous studies and generalize our conclusions to more severely impaired people who are often excluded from research.

### Limitations

The inclusive design comes with the drawback of limited formal data quality and the necessity to limit this pilot study to a more constrained procedure. First, we did not include repeated gaming sessions. Therefore, we cannot provide information on the potential long-term effects of the SGs. Second, self-reported data may be inaccurate given limited introspective abilities and difficulties understanding questions, potentially reducing the reliability of the results. We attempted to overcome this limitation by adding observational data and usage data. However, there is some potential for researcher bias in observational data since the assessment of state variables (eg, mood) in patients with dementia generally lacks reliability [51]. We took measures to ensure high-quality observational data by providing scoring guidelines and supervising the ratings done by investigators. Third, the number of study completers in the psychiatric ward was rather small in absolute terms, although it was average for this highly vulnerable population. Therefore, inferential tests must be interpreted with caution. Fourth, the control condition differed from the experimental conditions in more than one way (medium and content). For future research endeavors, it would be preferable to add analog versions of the SGs or a digital version of the newspaper to isolate the effects related to these attributes.

### Future Directions

Based on the promising findings from this pilot trial, it would be worthwhile to pursue a more extensive study design with a larger sample size, more recruitment sites, and repeated or longer gaming sessions, as well as repeated assessments over a longer period of time. Future studies should systematically register potential confounding factors that may influence usability at baseline (ie, sensory and motor impairment, and familiarity with technology) and incorporate the perspectives of caregivers and clinical staff. Future studies in the field would also benefit from establishing standards for quantifying the difficulty of SGs to allow the comparison of performance parameters within and across studies. This would help to evaluate the exact support requirements adapted to different profiles of impairment. Studying “people with dementia” without further specification is not sufficient for this aim, as has already been noted by Eichhorn et al [28].

### Conclusions

From the quantitative results presented herein, we can conclude that users with a wide variety of cognitive impairments were able to adequately use the SGs under the premise of adequate support. They reported good global usability, reported positive mood after playing, and showed a moderate error rate. Since a decrease in support could be seen from the first to the second usage of each SG, we can conclude that the games were sufficiently learnable.

The qualitative results underlined the importance of a user-centered design, considering cognitive as well as somatosensory impairments. In this regard, the adaptive difficulty levels represented an important step toward personalized care. Less impaired patients reached higher levels, contributing to the broad applicability of the SGs. Nevertheless, additional levels at both the low and high ends of difficulty would have been worthwhile. No significant differences in usability parameters were found between recruitment settings. SGs may therefore be suitable for not only people with MCI or MD but also those with severe cognitive impairment who are treated in an inpatient psychiatric setting. However, this requires overcoming practical restraints like quick patient turnover.

## Supplementary material

10.2196/69812 Multimedia Appendix 1 Need for support (yes or no) in the full-support run versus the minimal-support run.

## Abbreviations

CLMM: cumulative link mixed model
MCI: mild cognitive impairment
MD: mild dementia
MMSE: Mini-Mental State Examination
NPT: nonpharmaceutical treatment
SG: serious game
SUS: System Usability Scale
