# Supplementary figures and images for: Development of a Serious Game App (Digimenz) for Patients With Dementia: Prospective Pilot Study for Usability Testing in Inpatient Treatment and Long-Term Care
Source: JMIR Serious Games. 2025 Oct 27;13:e69812. doi: 10.2196/69812 (PMC12558423; doi:10.2196/69812)

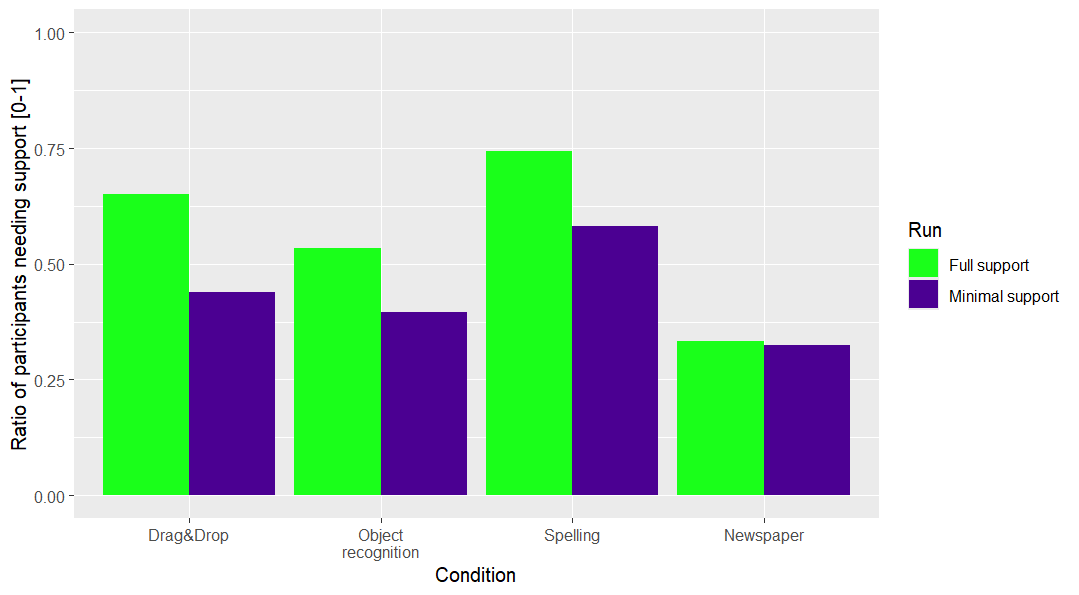

Supplement: Multimedia Appendix 1 [file games-v13-e69812-s001.png]
